# Prevalence and plasma exosome-derive microRNA diagnostic biomarker screening of adolescent idiopathic scoliosis in Yunnan Province, China

**DOI:** 10.3389/fped.2024.1308931

**Published:** 2024-04-24

**Authors:** Ping Yuan, Zhi-Hua Wang, Hong Jiang, Yang-Hao Wang, Jian-Yi Yang, Lu-Ming Li, Wen-Tong Wang, Jing Chen, Deng-Hui Li, Sheng-Yu Long, Wan Zhang, Fei He, Wei-Zhou Wang

**Affiliations:** ^1^ Department of Orthopedics, The First Affiliated Hospital of Kunming Medical University, Kunming, Yunnan, China; ^2^ The First Clinical College, Kunming Medical University, Kunming, Yunnan, China; ^3^ Trauma Medicine Centre, The First Affiliated Hospital of Kunming Medical University, Kunming, Yunnan, China; ^4^ Department of Medical Imaging, Kunming Children’s Hospital, Kunming Medical University, Kunming, Yunnan, China; ^5^ Department of Pathology, The First Affiliated Hospital of Kunming Medical University, Kunming, Yunnan, China; ^6^ Department of Orthopaedics, Kunming Guandu District People’s Hospital, Kunming, Yunnan, China; ^7^ Department of Orthopedics, Yunnan Sino-German Orthopedic Hospital, Kunming, Yunnan, China; ^8^ Department of Pathology and Pathophysiology, Faculty of Basic Medical Science, Kunming Medical University, Kunming, Yunnan, China; ^9^ Department of Orthopedic, Qujing Affiliated Hospital of Kunming Medical University, Qujing, Yunnan, China

**Keywords:** idiopathic scoliosis, children and adolescents, Yunnan region, school scoliosis screening, plasma exosome-derived miRNA, biomarkers

## Abstract

**Background:**

Idiopathic scoliosis significantly affects the physical and mental health of children and adolescents, with varying prevalence rates in different regions. The occurrence of idiopathic scoliosis is associated with genetic regulation and biochemical factors, but the changes in exosome-derived miRNA profiles among idiopathic scoliosis patients remain unclear. This study aimed to determine the prevalence of idiopathic scoliosis in Yunnan Province, China, and identify key exosome-derived miRNAs in idiopathic scoliosis through a cohort study.

**Methods:**

From January 2018 to December 2020, a cross-sectional study on idiopathic scoliosis in children and adolescents was conducted in Yunnan Province. A total of 84,460 students from 13 cities and counties in Yunnan Province participated in a scoliosis screening program, with ages ranging from 7 to 19 years. After confirmation through screening and imaging results, patients with severe idiopathic scoliosis and normal control individuals were selected using propensity matching. Subsequently, plasma exosome-derived miRNA sequencing and RT-qPCR validation were performed separately. Based on the validation results, diagnostic performance analysis and target gene prediction were conducted for differential plasma exosome-derived miRNAs.

**Results:**

The overall prevalence of idiopathic scoliosis in children and adolescents in Yunnan Province was 1.10%, with a prevalence of 0.87% in males and 1.32% in females. The peak prevalence was observed at age 13. Among patients diagnosed with idiopathic scoliosis, approximately 12.8% had severe cases, and there were more cases of double curvature than of single curvature, with thoracolumbar curvature being the most common in the single-curvature group. Sequencing of plasma exosome-derived miRNAs associated with idiopathic scoliosis revealed 56 upregulated and 153 downregulated miRNAs. Further validation analysis confirmed that hsa-miR-27a-5p, hsa-miR-539-5p, and hsa-miR-1246 have potential diagnostic value.

**Conclusions:**

We gained insights into the epidemiological characteristics of idiopathic scoliosis in Yunnan Province and conducted further analysis of plasma exosome-derived miRNA changes in patients with severe idiopathic scoliosis. This study has provided new insights for the prevention and diagnosis of idiopathic scoliosis, paving the way for exploring clinical biomarkers and molecular regulatory mechanisms. However, further validation and elucidation of the detailed biological mechanisms underlying these findings will be required in the future.

## 1 Introduction

Scoliosis is a three-dimensional spinal deformity characterized by a coronal curvature exceeding 10° and accompanied by vertebral rotation. Idiopathic scoliosis (IS) is the most common type of scoliosis, adolescent idiopathic scoliosis (AIS) referring to IS with onset between the ages of 10 and 18, accounting for approximately 90% of IS cases (1). AIS develops faster during adolescence. Without early detection and intervention, as the degree of deformity worsens, low back pain, impaired cardiorespiratory function, and nerve damage or even paraplegia eventually occur, severely affecting the physical and mental health of children and adolescents (2). Therefore, early detection and intervention is the current consensus in the treatment of AIS. The diagnosis and screening of AIS primarily rely on the patient's clinical appearance and x-ray images. However, the United States Preventive Services Task Force and the American Academy of Family Physicians recommend against routine scoliosis screening for asymptomatic adolescents due to its low specificity, potentially subjecting many low-risk adolescents to unnecessary x-rays and referrals (3–5). In clinical practice, when patients present with asymmetry in physical appearance, the best time for conservative treatment has often been missed. So, there is an urgent need to find new diagnostic markers for AIS to facilitate early screening for AIS.

The influence of factors such as region, ethnicity, and lifestyle, as well as differences in research methods and inclusion criteria, has led to significant variations in the prevalence of AIS (6). Globally, the prevalence of AIS ranges from approximately 0.47% to 5.2%. The prevalence is higher in females than in males, with female-to-male ratios ranging from 1.5 to 11 (7, 8). In China, the prevalence of AIS is reported to be between 0.11% and 2.6%, with most data originating from economically developed eastern and southern regions, while there is limited reporting of AIS prevalence in western China (9, 10). Yunnan, located on the southwestern border of China and situated on the Yunnan-Guizhou Plateau, is home to 26 different ethnic groups, making it the province with the most diverse national minority in China. As a result, the prevalence of scoliosis in this region may differ. But the epidemiological characteristics of AIS among primary and secondary school students in this area remain unclear.

AIS arises from complex interactions between genetic and environmental factors, and these interactions are mediated through integrated biological and biomechanical mechanisms (7). Environmental, nutritional, and lifestyle factors can modulate the epigenome, thereby promoting AIS progression (11). Consequently, epigenetics holds the potential to provide new biomarkers for the diagnosis and prognosis of AIS, aiding in the analysis of the molecular factors underlying the disease. RNA serves as a vital epigenetic regulatory entity. Studies have demonstrated that the expression levels of key messenger RNAs, microRNAs (miRNAs), or long noncoding RNAs are associated with the height of AIS patients, and their expression varies among different developmental stages, Cobb angles, and Risser grades (12–15). Exosome-derived miRNAs in peripheral blood are considered significant contributors to osteogenesis and bone metabolism. They are closely associated with intervertebral disc degeneration. This makes them intriguing biological molecules for investigating the causes of AIS (16–19). However, research on exosome-derived miRNAs in AIS is still in its nascent stage.

This study was based on the results of school scoliosis screening (SSS) conducted in 13 regions of Yunnan Province, China, involving children and adolescents aged 7–19 years. The aim was to investigate the characteristics and prevalence of AIS in Yunnan Province and to provide more theoretical basis for the control of AIS in Yunnan Province. Additionally, a portion of AIS patients and age-matched individuals with normal spine conditions were selected from the screened population, and their plasma samples were collected for the sequencing and bioinformatics analysis of AIS-related peripheral blood exosome-derived miRNAs. Real-time quantitative PCR (RT-qPCR) was employed to validate the expression of these exosome-derived miRNAs, laying the foundation for exploring early AIS-specific molecular markers and potential mechanisms.

## 2 Methods

### 2.1 Study design and subjects

This study was divided into two phases. The first phase involved a cross-sectional SSS study in children and adolescents in Yunnan Province. It was conducted in school health clinics from January 2018 to December 2020. The study covered 13 different regions in Yunnan Province, including Kunming City, Zhaotong City, Shangri-La City, Yuxi City, Chuxiong City, Dali Prefecture, Wenshan Prefecture, Honghe Prefecture, Qujing City, Pu'er City, Lijiang City, Lincang City, and Tengchong City. Primary school students, junior high school students, high school students, and students enrolled in vocational high schools from different schools in the 13 cities and municipalities were randomly selected as the study subjects, and the age range of the participants in the survey was mainly 7–19 years.

The second phase involved radiological assessment and the screening of AIS plasma markers. All subjects identified as potentially having scoliosis through the initial screening were referred to local medical facilities for radiological assessments. Upon completion of the imaging assessment and regular follow-up, peripheral blood was collected for study use if the subject met the inclusion and exclusion criteria for plasma exosomal molecular marker screening. The inclusion criteria for plasma exosomal molecular marker screening comprised the following: individuals diagnosed with AIS, with a Cobb angle greater than 40°, at risk of progressive Cobb angle increase during follow-up, without prior surgical treatment, and aged between 10 and 18 years. Exclusion criteria included bad habits such as smoking, the occurrence of active infections or inflammatory diseases within the last one month, long-term use of drugs affecting bone metabolism, neurological pathologies, congenital developmental anomalies or nutritional deficiencies, and a history of tumours. Students who did not provide consent to participate in the study either themselves or through their guardians were also excluded. Additionally, a subset of adolescents aged 10–18 years who were physically healthy and free of AIS were also recruited as a control group, and their peripheral blood was collected for the study.

The study was approved by the schools in the surveyed area and the Medical Research Ethics Committee of the First Affiliated Hospital of Kunming Medical University (2022-L152). Parents or guardians provided consent for students to participate in this study before the students completed the surveys and underwent imaging assessments. All the subjects in this study provided written consent for their samples and related information to be used in this study. All experimental procedures, protocols and methods were in accordance with relevant clinical guidelines and regulations, following standard operating procedures.

### 2.2 Phase one screening methodology and procedures

Prior to their participation in the SSS, uniform training was provided to all screening inspectors. The screening process primarily consisted of two steps. First, students underwent the Adam Forwards Bend Test to observe whether they exhibited any signs of chest asymmetry, shoulder blade asymmetry, waist asymmetry, pelvic tilt, or deviation of the spinous process line from the midline (20). Students who were positive for one of these were further examined for angle of trunk rotation (ATR) (6). Following the guidelines established by the International Society on Scoliosis Orthopaedic and Rehabilitation Treatment (21) and the Screening Criteria for Scoliosis in China (GB/T 16133–2014) (6), students with an ATR ≥ 5° were considered to have suspected positive results in the scoliosis screening. They were subsequently referred to a hospital for confirmation of the condition through full-length spinal x-ray examinations.

### 2.3 Phase two screening methodology and procedures

For students with suspected positive results, anterior-posterior and lateral x-ray images of the spine were taken, and the Cobb angle was measured using the standard Cobb method. Students with a Cobb angle ≥10° were diagnosed with scoliosis. Information such as age, sex, ethnicity, family history, place of origin, educational stage, and anthropometric measurements (including height, weight, and sitting height) was collected for students who were confirmed through radiological methods. Additionally, information on neurological, muscular, or skeletal conditions was gathered to exclude congenital scoliosis, neuromuscular scoliosis or other related conditions. Students with a Cobb angle ≥10° and <20° were advised to undergo regular observation, while those with a Cobb angle ≥20° and <40° were recommended to undergo rehabilitation exercises or wear orthotic devices under the guidance of healthcare professionals. Students with a Cobb angle ≥40° were advised to undergo surgical treatment (22). Selected AIS subjects and healthy individuals who met the inclusion exclusion criteria for the plasma exosomal molecular marker screening study were recruited at this stage. Peripheral blood was collected for subsequent studies after signing an informed consent form.

### 2.4 Peripheral blood plasma collection and study subgroups

For volunteers providing peripheral blood, 10 ml of fasting peripheral venous blood was collected in EDTA-K2 anticoagulant tubes. After collection, samples were centrifuged at 3,500 rpm for 5 min using a low-speed refrigerated centrifuge (5702R, Eppendorf, Germany) to remove blood cells and platelets, and 3–4 ml of the plasma supernatant was collected. Purified samples were then labelled and stored in liquid nitrogen. For all the collected plasma samples, the AIS group and the normal control group were matched one-to-one based on age, sex, and ethnicity using propensity score matching. After grouping, one group was randomly selected for the isolation and identification of plasma exosomes, while six other groups underwent high-throughput sequencing of exosome-derived miRNAs. The remaining groups were subjected to RT-qPCR validation based on the sequencing results.

### 2.5 Plasma exosome isolation and purification

The 4 ml plasma samples were rapidly thawed at 37°C. Exosome isolation and purification were performed using the Plasma/Serum Exosome Purification Kit (Qiagen, Norgen Biotek, Canada) following the manufacturer's instructions. Initially, the plasma was diluted by adding 12 ml of nuclease-free water, followed by the addition of 300 μl of ExoC Buffer and 400 μl of Slurry E. After being incubated at room temperature for 5 min, the mixture was centrifuged at 2,000 revolutions per minute (RPM) for 2 min to remove the supernatant. Subsequently, 400 μl of ExoR Buffer was added, and the mixture was incubated for 10 min. Afterwards, it was centrifuged using a spin column at 6,000 RPM for 1 min, resulting in purified exosomes.

### 2.6 Exosome identification

The purified exosomes were subjected to transmission electron microscopy, size analysis, immunofluorescence and nanoflow cytometry detection. For transmission electron microscopy, 10 μl of exosomes was dropped onto a copper grid, allowed to settle for 1 min, and then stained with uranyl acetate (Sigma-Aldrich, USA). After incubation and drying, the exosomes were imaged using a Hitachi HT-7700 transmission electron microscope at 100 kV. A 10 μl aliquot of exosome sample was initially diluted to 30 μl. Subsequently, the exosome size was analysed using an N30E size analyser (NanoFCM, China). A 30 μl aliquot of diluted exosome sample was mixed with 20 μl of fluorescently labelled antibodies, including CD9 (FITC Mouse Anti-Human CD9, BD) and CD81 (FITC Mouse Anti-Human CD81, BD). The mixture was incubated in the dark for 30 min. Subsequently, it was subjected to ultracentrifugation at 110,000 ×g and 4°C for 70 min twice to obtain the supernatant. Afterwards, the exosomes were resuspended in 50 μl of PBS and analysed using a nanoscale flow cytometer (NanoFCM, China).

### 2.7 Plasma exosome-derived miRNA sequencing

After propensity matching, a total of 6 sample groups were selected (AIS patients = 6, healthy individuals = 6) for exosome-derived miRNA sequencing analysis. Following sample thawing, exosomes were purified using the Plasma/Serum Exosome Purification Kit (Qiagen, Norgen Biotek, Canada). Exosome-derived miRNA was extracted using the Exosomal RNA Isolation Kit (Qiagen, Norgen Biotek, Canada) according to the manufacturer's protocol. The extracted exosome-derived miRNA was then amplified. TruSeq Small RNA Sample Prep Kits (Illumina, San Diego, USA) were employed for library preparation. Sequencing was carried out using the Illumina Hiseq2000/2500 platform with a single-end sequencing read length of 1 × 50 bp.

### 2.8 Analysis of sequencing results

After sequencing was completed, the data were analysed using the miRNA data analysis software ACGT101-miR (LC Sciences, Houston, Texas, USA). The analysis process was as follows: the raw data were processed by quality control to obtain clean reads, and the 3' junctions were removed from the clean reads and screened based on length, retaining sequences with base lengths of 18–26 nt. The remaining sequences were filtered against the mRNA database, RFam database and Repbase database, and the non-miRNA components, such as ribosomal RNA, transfer RNA, mini-RNA and minicellular RNA, were excluded, while the remaining were considered valid data. Then, the valid data from the AIS and normal groups were compared, and the exosome-derived miRNAs with *P**** ***< 0.05 and with 2-fold higher or lower expression in each AIS sample than in the matched normal sample were selected as the candidate differential exosome-derived miRNAs.

### 2.9 Validation of candidate exosome-derived miRNAs by RT-qPCR

Candidate exosome-derived miRNAs were subjected to RT-qPCR validation analysis using additional samples from the AIS group (*n* = 23) and the normal group (*n* = 23). Exosome-derived miRNA extraction was performed using the method described above. The NovoScript miRNA First-Strand cDNA Synthesis and SYBR qPCR Kit (Qiagen, Novoprotein, Japan) was utilized following the manufacturer's instructions for first-strand cDNA synthesis and qPCR amplification. A mixture containing 1 μg of miRNA samples, 10 μl of 2× miRNA RT Reaction Mix, 1 μl of NovoScript miRNA RT Enzyme Mix, and 9 μl of nuclease-free water was prepared, with a final volume of 20 μl. The mixture was incubated at 39°C for 60 min, followed by 5 min at 85°C to complete first-strand cDNA synthesis. Two-step amplification was performed using the ABI PRISM 7500 sequence detection system (Applied Biosystems, Bedford, Massachusetts, USA). Each PCR was performed in triplicate. Primer sequences and reference sequences are provided in Supplementary Table S1. The relative expression levels of exosome-derived miRNAs were calculated using the 2^−ΔΔCT^ method.

### 2.10 Target gene analysis and statistical analysis of pathway and functional enrichment

The significantly different exosome-derived miRNAs were subjected to target gene prediction using two software programs: TargetScan (v5.0) (23–25) and miRanda (v3.3a) (26–28). Target genes predicted by each software were filtered according to their respective scoring criteria. For the TargetScan algorithm, target genes with context score percentiles below 50 were excluded. For the miRanda algorithm, target genes with a Max Energy greater than −10 were removed. The final target genes of different exosome-derived miRNAs were selected as the intersection of the predictions from these two software programs (i.e., the threshold was set as TargetScan_score ≥ 50 and miranda_Energy < −10). Gene Ontology (GO) enrichment and Kyoto Encyclopedia of Genes and Genomes (KEGG) enrichment analyses were conducted on the identified target genes.

### 2.11 Statistical analysis

The data from the first stage of the study were entered into a database file using Microsoft Excel 2021, including all questionnaire information, radiological assessment results, and epidemiological survey results. Statistical analysis and result visualization were performed using GraphPad Prism v9.5.0 (GraphPad Software Inc., San Diego, CA). Differences in height, weight, and sitting height between the AIS group and the normal group were compared using independent sample *t* tests or nonparametric tests. Pearson's chi-squared test (*χ*^2^) was used to assess the differences in BMI and educational stage between the AIS and normal groups. *χ*^2^ or Fisher's exact tests were used to compare AIS prevalence among different age groups and sexs, as well as differences in spinal deformity among different AIS subgroups. *P* < 0.05 was considered indicative of statistical significance.

In the second stage of the study, the sequencing results data were first normalized, and the normality of the data was assessed. When biological replicates were present, differences between two sample groups were analysed using the t test, and differences among multiple groups were analysed using ANOVA. When there were no biological replicates, differences between two sample groups were assessed using Fisher's exact test and *χ*^2^, while differences among multiple groups were assessed using *χ*^2^. For RT-qPCR results, differences between two sample groups were calculated using the *t* test or nonparametric tests. The differences between the two sample groups in RT-qPCR results were calculated using either the t test or nonparametric tests. The area under the receiver operating characteristic curve (ROC) was used to evaluate the predictive efficiency of differential exosome-derived miRNAs. All statistical analyses and result visualizations were performed using the OmicStudio tools (at: https://www.omicstudio.cn/tool) or GraphPad Prism v9.5.0 (GraphPad Software Inc., San Diego, CA). *P* < 0.05 was considered indicative of statistical significance.

## 3 Results

### 3.1 Scoliosis screening summary

**Figure 1:**
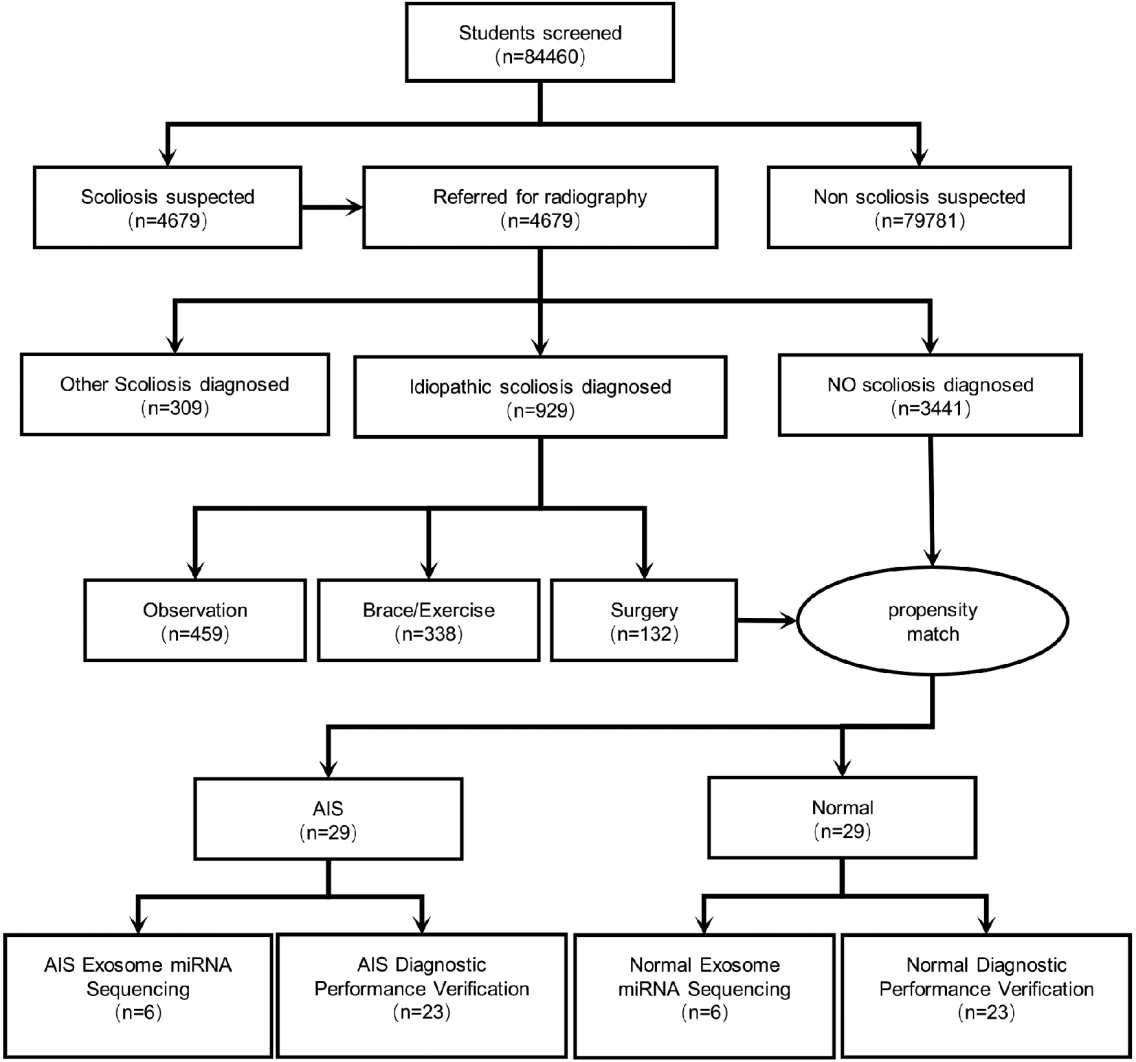
Study workflow diagram.

In this study, a total of 84,460 children and adolescents aged 7–19 years underwent scoliosis screening. These individuals included 41,115 males and 43,345 females, with a male-to-female ratio of 1:1.2. Among the screened individuals, 4,679 (5.54%) were suspected of having scoliosis and underwent radiographic examination and reassessment. Among them, 929 (1.10%) were diagnosed with IS, and 309 (0.37%) were diagnosed with other types of scoliosis. Ultimately, among the confirmed IS patients, 459 (49.41%) were recommended for observation, 338 (36.38%) were advised to undergo rehabilitation via exercise or orthotic wear, and 132 (14.21%) required surgical intervention. Blood plasma samples were collected from patients requiring surgical intervention and from individuals without scoliosis. Subsequently, propensity score matching was performed to obtain 29 sets of AIS samples and normal samples with matched age, sex, and ethnicity. Six groups underwent high-throughput sequencing of exosome-derived miRNAs, while the remaining 23 groups were used for the validation of the diagnostic efficacy of candidate differential exosome-derived miRNAs (Figure 1).

### 3.2 Prevalence of scoliosis stratified by age

The prevalence of IS among the 929 patients was compared by grouping them according to sex and age. The results, as shown in Table 1, illustrate the disease prevalence among different age and sex groups. The overall prevalence rate among males was 0.87%, while among females, it was 1.32%. The overall prevalence rate among females was significantly higher than that among males (*P* < 0.05). The prevalence was higher among females than males at all ages. However, there was no significant difference between the prevalence rates of males and females at ages 7–11 years (*P**** ***> 0.05), and the difference was significant at ages 12–16 years (*P**** ***< 0.05). At age 13, there was a peak in the prevalence of IS among females (2.54%) and males (1.56%). Subsequently, the prevalence rates gradually decreased in both genders, and by the age of 17, there was no statistically significant difference in the prevalence rates between males and females (*P**** ***> 0.05). At the peak of the disease prevalence, females had a prevalence rate 1.62 times higher than that of males.

**Table 1:** Prevalence of IS by age and gender.

Age	Male			Female			Chi-square	*P*
	Total number	Scoliosis screening positive	Positive rate %	Total number	Scoliosis screening positive	Positive rate %		
7	3,093	6	0.19	3,128	8	0.26	0.264	0.514
8	2,831	5	0.18	2,970	5	0.17	0.005	0.939
9	3,025	9	0.30	3,540	13	0.37	0.237	0.626
10	3,283	20	0.61	3,432	28	0.82	1.010	0.315
11	3,697	20	0.54	3,834	34	0.89	3.162	0.0754
12	3,196	40	1.25	3,294	74	2.25	9.305	0.002
13	3,077	48	1.56	3,234	82	2.54	7.439	0.006
14	3,152	47	1.49	3,352	84	2.51	8.477	0.003
15	2,955	43	1.46	3,011	65	2.16	4.154	0.041
16	3,178	46	1.45	3,525	82	2.33	6.890	0.008
17	3,531	40	1.13	3,570	51	1.43	1.227	0.267
18	3,371	19	0.56	3,574	28	0.78	1.247	0.264
19	2,726	14	0.51	2,881	18	0.62	0.305	0.581
Total	41,115	357	0.87	43,345	572	1.32	39.51	0.0001

### 3.3 Participant demographic characteristics and is-related factors

The population characteristics of the participants and the risk factors associated with IS are detailed in Table 2. When comparing the IS group to the normal group, there were no statistically significant differences in height, but there were statistically significant differences in weight and sitting height (*P* < 0.05). In the IS group, 477 individuals (51.35%) had a BMI less than 18.5, while in the normal group, 43,708 individuals (52.52%) had a BMI less than 18.5, with no statistically significant difference between the two groups. Among students attending primary school, junior high school, and high school, there were 262 cases (28.20%), 369 cases (39.72%), and 298 cases (32.08%) of IS, respectively, with the highest prevalence among junior high school students, with a significant difference (*P* < 0.0001).

**Table 2:** Analysis of participants’ demographic characteristics and risk factors.

Variant	IS (*n* = 929)	Normal (*n* = 83,222)	Chi-square/*t*	*P*
Hight (cm)*	156.53 ± 14.45	156.84 ± 14.95	0.650	0.5158
Sitting height (cm)*	82.48 ± 7.78	83.23 ± 7.57	2.923	0.0036
Weight (kg)*	46.69 ± 10.91	47.35 ± 10.33	1.835	0.0669
BMI (*n*, %)				
≥18.5	496 (53.39)	43,752 (52.57)	0.2466	0.6195
<18.5	433 (46.61)	39,470 (47.43)		
Education level (*n*, %)				
Primary School	262 (28.20)	38,915 (46.76)	197.4	0.0001
Junior High School	369 (39.72)	18,342 (22.04)		
Senior High School	298 (32.08)	25,965 (31.20)		

IS* data are expressed as the average ± SD.

### 3.4 Characteristics of the spinal deformities in is patients

**Figure 2:**
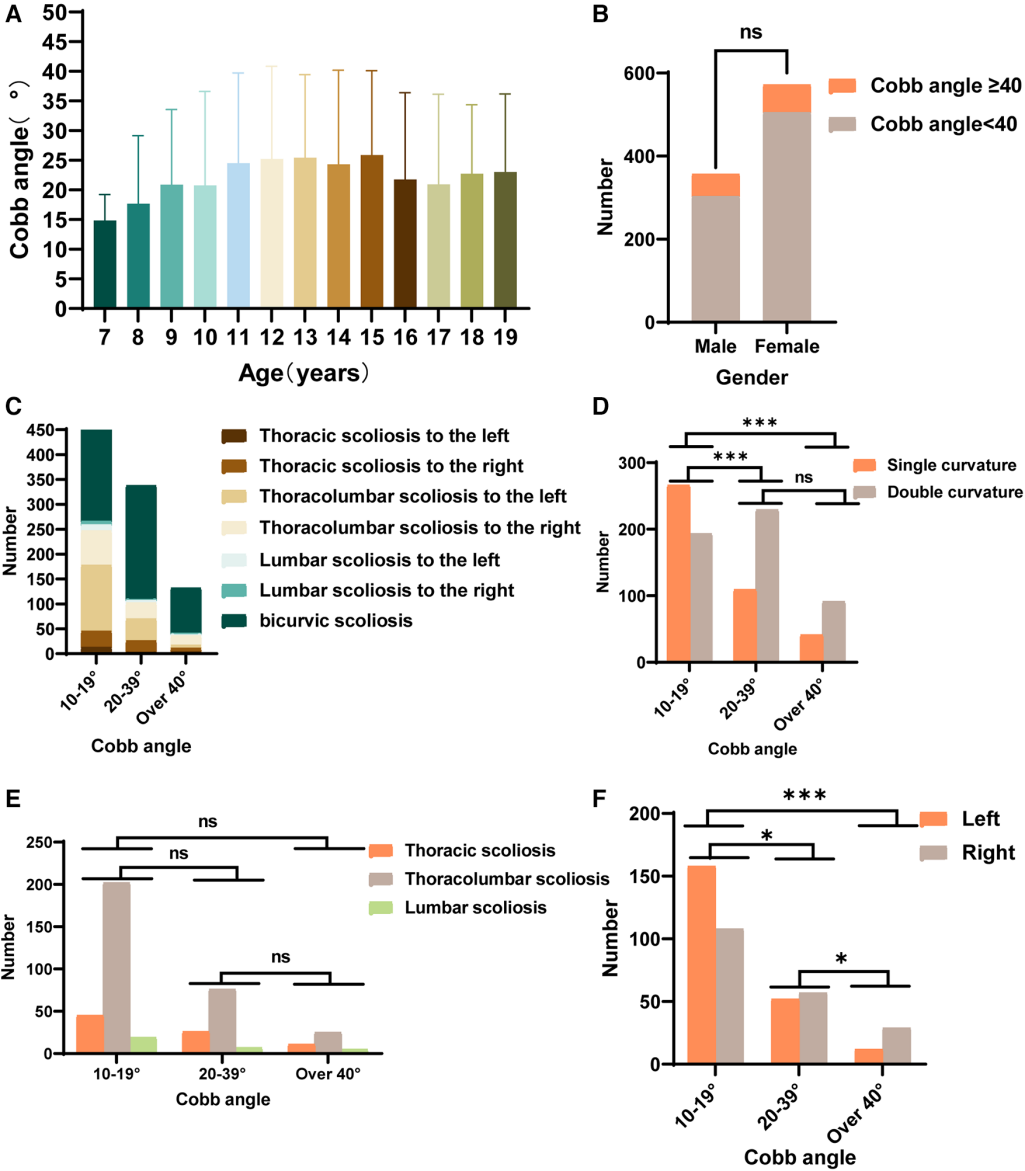
Statistics of spinal deformities in IS patients. (**A**) The distribution of Cobb angles in IS patients across different age groups. (**B**) The number of males and females with Cobb angles ≥ 40° among IS patients. (**C**) The distribution of spinal deformity types in IS patients with mild, moderate, and severe curvatures. (**D**) Statistics for the number of individuals with double curvature and single curvature among IS patients with mild, moderate, and severe curvatures. (**E**) Statistics for the number of patients with thoracic curvature, thoracolumbar curvature, and lumbar curvature among IS patients with mild, moderate, and severe curvatures. (**F**) Statistics for the number of IS patients with main spinal curves showing left or right convexity among those with mild, moderate, and severe curvatures. (ns *P *> 0.05, *P < 0.05, **P*** ***< 0.001, ***P < 0.0001).

The prevalence and number of Cobb angles varied by age group. Cobb angles were determined in IS patients of all ages, and it was found that the mean Cobb angle was larger in 13- and 15-year-old patients (Figure 2A). Among the 929 scoliosis patients, 121 individuals were classified as having severe scoliosis (Cobb angle ≥ 40°), resulting in a detection rate of 12.80%. The male-to-female ratio was approximately 9:11, and there was no statistically significant difference in the prevalence of severe IS between sexs (Figure 2B). Analysis of the curvature deformities was conducted, and the results indicated that among the 929 IS patients, the most common type of curvature deformity was double major curves. When specifically examining the cases with single-curvature scoliosis, it was observed that the majority of mild cases (10° < Cobb angle < 20°) and moderate cases (20° ≤ Cobb angle < 40°) had left convex thoracolumbar curves. Among the severe cases, the most prevalent type of curvature deformity was right convex thoracolumbar curves (Figure 2C). The number of patients with single- or double-curvature scoliosis differed between the mild group and the moderate and severe groups and did not differ between the moderate and severe groups (Figure 2D). Among patients with single-curvature scoliosis, most had thoracolumbar curvatures, followed by thoracic curvatures, with lumbar curvatures being the least common, and there was no difference in the distribution of curvatures between the groups (Figure 2E). Among mild single-curvature cases, left convex curves were more common than right convex curves. In moderate single-curvature cases, left convex curves were slightly less common than right convex curves, and in severe single-curvature cases, left convex curves were significantly less common than right convex curves. Statistically significant differences were observed among all groups in terms of curve convexity (Figure 2F).

### 3.5 Clinical profile of patients with AIS

In all the collected peripheral blood samples, AIS plasma samples and normal control plasma samples were paired using propensity matching to minimize bias in the results due to age, sex, and ethnicity. This matching process resulted in 29 sets of samples. Six sets were randomly selected for sequencing, while the remaining 23 sets underwent RT-qPCR validation. There were no differences in the corresponding human measurement parameters (BMI, family history, and Risser sign), as shown in Table 3.

**Table 3:** Subject information.

Variant	Exosome-derived miRNA sequencing groups			RT-qPCR validation group		
	AIS (*n* = 6)	Normal (*n* = 6)	*P*	AIS (*n* = 23)	Normal (*n* = 23)	*P*
Age^a,b^ (year)	11.83 ± 2.14	11.83 ± 2.14	–	15.13 ± 2.26	15.13 ± 2.26	–
Male/Female^a,b^	4/2	4/2	–	6/17	6/17	–
BMI (kg/m^2^)	18.60 ± 3.37	18.87 ± 2.39	0.878	18.83 ± 3.11	18.84 ± 3.19	0.9870
Family history	No	No	–	No	No	–
Cobb angle (°)	66.17 ± 15.52	0	–	52.04 ± 19.93	0	–
Risser sign	0.83 ± 1.33	0.67 ± 1.03	0.999	2.87 ± 1.66	2.78 ± 1.62	0.7835

^a^ Represents the information used for propensity matching, which was the same between the two groups.

^b^ Values are shown as averages ± SD.

### 3.6 Plasma exosome characteristics

**Figure 3:**
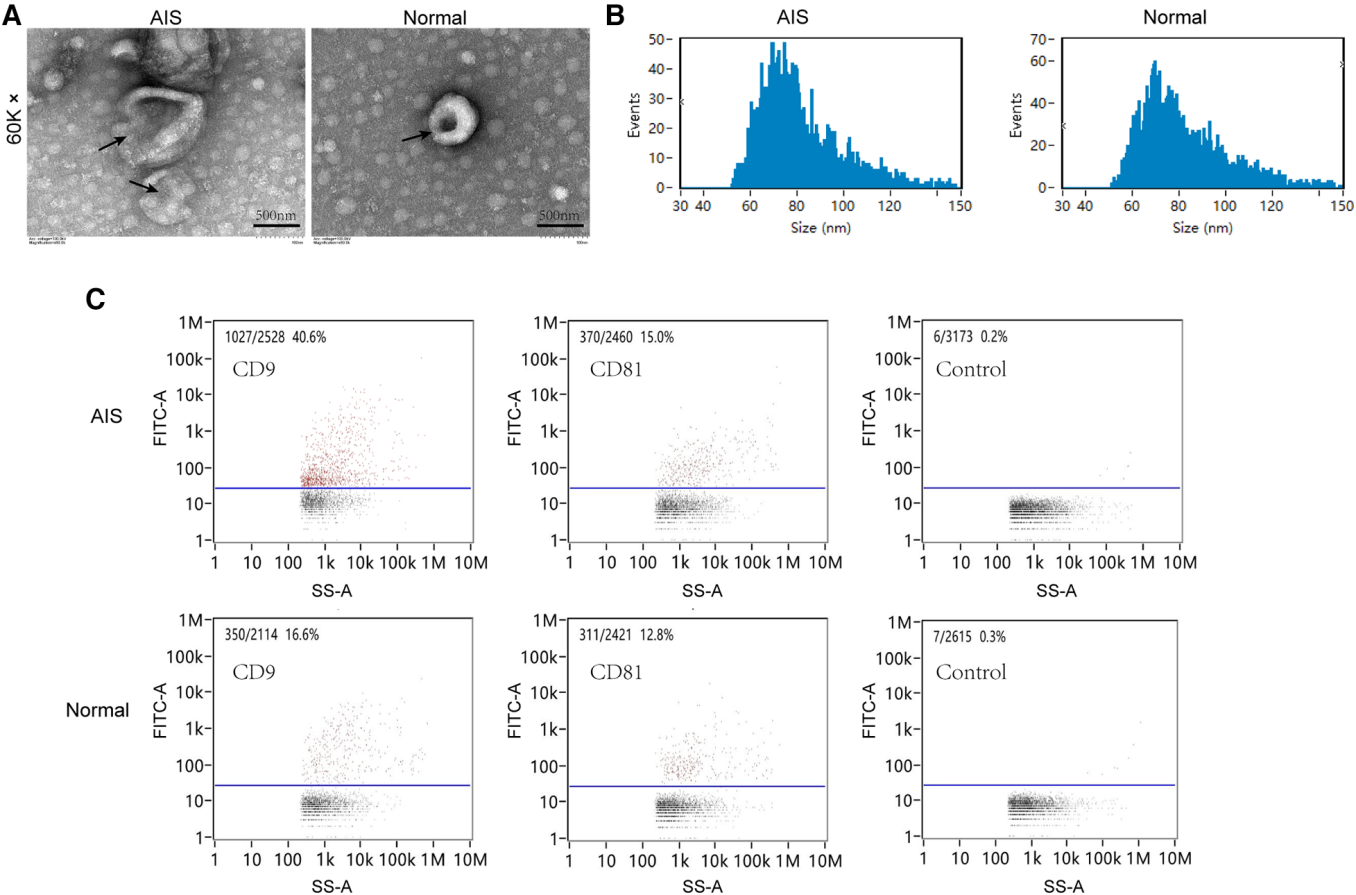
Plasma exosome characterization. (**A**) Transmission electron microscopy scanning of plasma exosomes from AIS and normal samples. (**B**) Particle size analysis of plasma exosomes in AIS and normal samples. (**C**) Nano flow cytometry analysis of CD9/CD81 in plasma exosomes from AIS and normal samples.

This study analysed the characteristics of plasma exosomes in AIS patients. Exosomes were extracted using a centrifugation column method, and the morphology of isolated exosomes was observed using transmission electron microscopy. Both groups of exosomes exhibited a spherical vesicular shape (Figure 3A). The particle size analysis results (Figure 3B) showed that the average particle size of exosomes in the AIS group was 81.83 nm, with a median particle size of 77.25 nm, while in the normal group, the average particle size of exosomes was 81.39 nm, with a median particle size of 77.25 nm. Nanoflow cytometry analysed the surface-tagged proteins CD9 and CD81 on exosomes from both sets of samples and found that both sets of samples expressed both proteins. The results of two randomly selected samples are shown below (Figure 3C).

### 3.7 Sequencing analysis of plasma exosome-derived miRNAs

**Figure 4:**
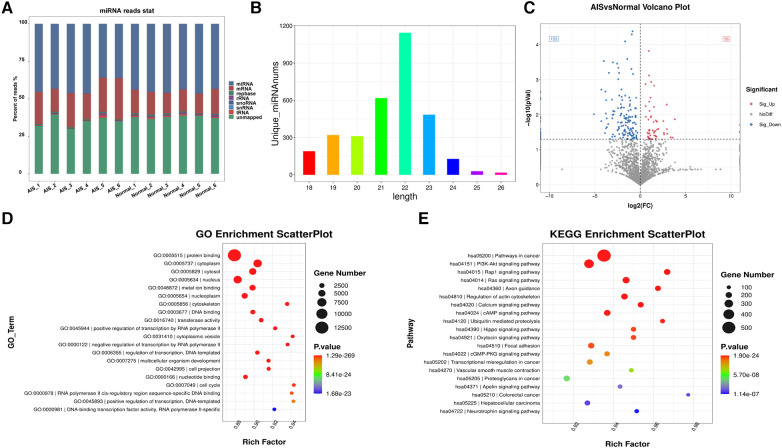
Exosome-derived miRNA sequencing. (**A**) Comparison of sequencing results with various databases. (**B**) Length distribution of exosome-derived miRNAs. (**C**) Differentially expressed exosome-derived miRNAs between the AIS group and the normal group (*P* < 0.05). (**D**) GO-enriched TOP20 bubble plot of differential exosome-derived miRNAs. (**E**) KEGG-enriched TOP20 bubble plot of differential exosome-derived miRNAs.

High-throughput RNA sequencing was employed to analyse the miRNA profiles of plasma exosomes in 6 propensity-matched AIS patients and 6 healthy controls. The obtained sequences were aligned and compared with the mRNA, RFam (which includes rRNA, tRNA, snRNA, and snoRNA sequences), and Repbase databases (Figure 4A). The percentage of identified exosome-derived miRNAs in AIS samples averaged 42.24%, while in the healthy control group, it averaged 44.84%. There was no significant difference between the two groups. The statistical analysis of the identified exosome-derived miRNA lengths (Figure 4B) revealed that the lengths were primarily concentrated between 21 and 23 base pairs, with the highest number of exosome-derived miRNAs having a length of 22 base pairs.

The miRNA expression levels were normalized using ACGT101-miR software, followed by differential expression analysis. A comparison of exosome-derived miRNA expression between the AIS group and the normal group was conducted, and a volcano plot was generated (Figure 4C). Compared to the normal group, there were 209 differentially expressed exosome-derived miRNAs in AIS patients (*P* < 0.05), with 56 upregulated and 153 downregulated miRNAs. Differential exosome-derived miRNAs were also compared between matched samples, and it was observed that the differential exosome-derived miRNAs were different in each group (Supplementary Figure S1).

The biological functions of all differentially expressed exosome-derived miRNAs in AIS patients (*P* < 0.05) were predicted for target genes, followed by GO and KEGG enrichment analyses. The GO enrichment results are shown in the bubble chart (Figure 4D). In the molecular function category, the most significantly enriched and gene-abundant function was related to protein synthesis. In the cellular component category, the most significantly enriched and gene-abundant component was the cytoplasm. In the biological process category, the most significantly enriched and gene-abundant process was positive regulation of transcription by RNA polymerase II. The top 20 KEGG enrichment results are shown in the bubble chart (Figure 4E). The cancer-related signalling pathway had the highest number of enriched genes and was significantly enriched. The enrichment level of the PI3K-Akt signalling pathway was slightly lower than that of the cancer pathway.

### 3.8 Plasma candidate-specific exosome-derived miRNA screening

**Figure 5:**
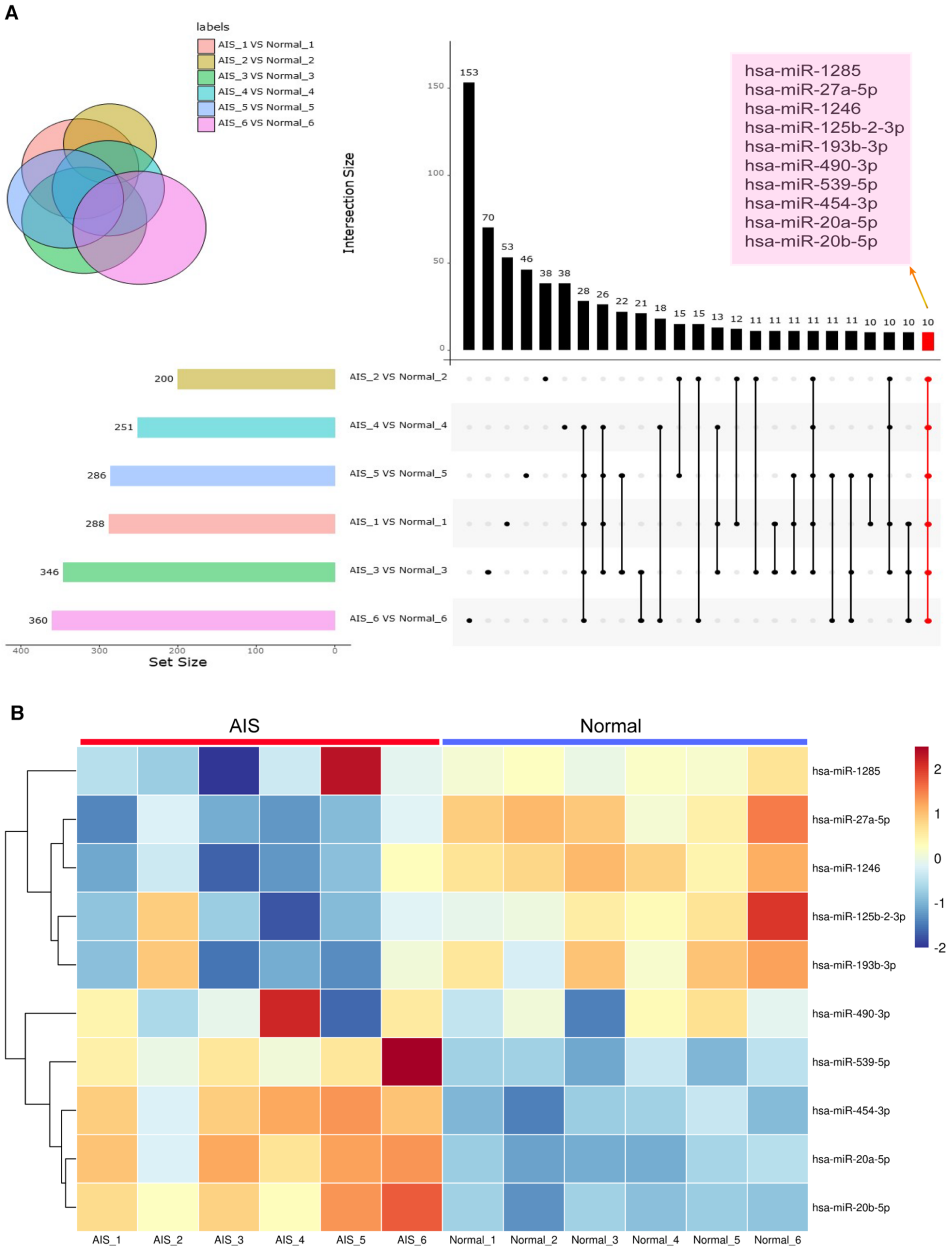
Exosome-derived miRNA analysis. (**A**) The setup figure compares differentially expressed exosome-derived miRNAs in the plasma exosomes of 6 AIS patients and their matched normal controls. The common differentially expressed exosome-derived miRNAs in all 6 groups are highlighted in coloured boxes. (**B**) The heatmap compares the expression levels of shared differentially expressed exosome-derived miRNAs in 6 AIS patients and 6 normal volunteers.

Comparison of differential exosome-derived miRNAs among the 6 groups and screening for shared differential exosome-derived miRNAs were performed (Figure 5A). A total of 10 miRNAs were screened in the 6 groups, namely, hsa-miR-1246, hsa-miR-125b-2-3p, hsa-miR-193b-3p, hsa-miR-20a-5p, hsa-miR-20b-5p, hsa-miR-27a-5p, hsa-miR-454-3p, hsa-miR-490-3p, hsa-miR-539-5p, and hsa-miR-1285-p5. Cluster analysis of the expression levels of these 10 differentially expressed exosome-derived miRNAs in each sample was performed (Figure 5B). Compared to the normal group, hsa-miR-125b-2-3p, hsa-miR-193b-3p, hsa-miR-490-3p, and hsa-miR-1285-p5 in AIS samples exhibited lower expression levels that were inconsistent with other samples. Hsa-miR-1246 and hsa-miR-27a-5p showed decreased expression in all AIS samples, while hsa-miR-20a-5p, hsa-miR-20b-5p, hsa-miR-454-3p, and hsa-miR-539-5p showed increased expression in all AIS samples. Therefore, hsa-miR-1246, hsa-miR-27a-5p, hsa-miR-20a-5p, hsa-miR-20b-5p, hsa-miR-454-3p, and hsa-miR-539-5p were selected as candidate AIS-specific exosome-derived miRNAs in plasma.

### 3.9 RT-qPCR validation of candidate exosome-derived miRNA diagnostic efficacy

**Figure 6:**
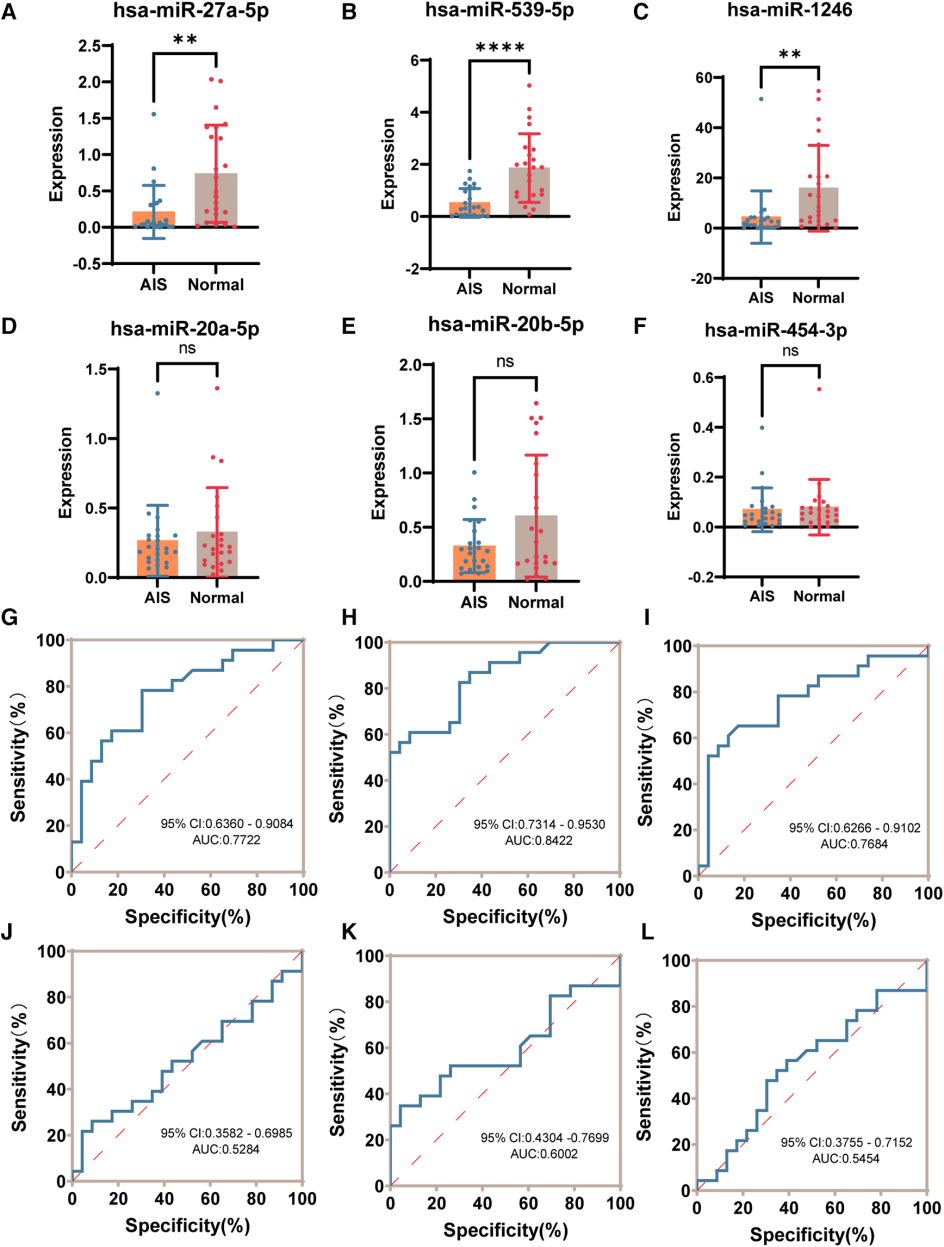
RT-qPCR validation of candidate differential exosome-derived miRNAs. (**A**) Expression of hsa-miR-27a-5p in the AIS and normal groups. (**B**) Expression of hsa-miR-539-5p in the AIS and normal groups. (**C**) Expression of hsa-miR-1246 in the AIS and normal groups. (**D**) Expression of hsa-miR-20a-5p in the AIS and normal groups. (**E**) Expression of hsa-miR-20b-5p in the AIS and normal groups. (**F**) Expression of hsa-miR-454-3p in the AIS and normal groups. (**G**) ROC curve of hsa-miR-27a-5p validating the diagnostic efficacy. (**H**) ROC curve of hsa-miR-539-5p to validate diagnostic efficacy. (**I**) ROC curve of hsa-miR-1246 validating the diagnostic efficacy. (**J**) ROC curve of hsa-miR-20a-5p validating the diagnostic efficacy. (**K**) ROC curve of hsa-miR-20b-5p validating the diagnostic efficacy. (**L**) ROC curve of hsa-miR-454-3p validating the diagnostic efficacy. (ns *P* > 0.05, *P < 0.05, **P < 0.005, ***P < 0.001, ****P*** ***< 0.0001).

Validation of the 6 candidate differentially expressed exosome-derived miRNAs (hsa-miR-1246, hsa-miR-27a-5p, hsa-miR-20a-5p, hsa-miR-20b-5p, hsa-miR-454-3p, hsa-miR-539-5p) in the plasma samples of 23 AIS patients and 23 normal volunteers was performed using quantitative RT-qPCR technology. Based on the sequencing results, hsa-miR-26a-5p exhibited high expression abundance in all samples and showed minimal differences among the groups. Therefore, it was chosen as an internal reference (29). After normalization using hsa-miR-26a-5p, the expression levels of 6 candidate plasma exosome-derived miRNAs were compared between peripheral blood from AIS patients and that from normal volunteers (Figures 6A–F). The results showed that compared to the normal group, AIS patients had significantly downregulated expression of hsa-miR-27a-5p, hsa-miR-539-5p, and hsa-miR-1246 in plasma exosomes (*P* < 0.05). However, there was no statistically significant difference in the expression of hsa-miR-20a-5p, hsa-miR-20b-5p, or hsa-miR-454-3p between the two groups (*P* > 0.05). Then, ROC curves were constructed using the normalized 2^−ΔΔCT^ values to validate the diagnostic efficacy of these differentially expressed exosome-derived miRNAs for AIS (Figures 6G–L). The results showed that low expression of hsa-miR-27a-5p, hsa-miR-539-5p, and hsa-miR-1246 was able to successfully differentiate AIS patients from healthy individuals, with AUCs of 0.7722 (95% CI: 0.6360–0.9084, sensitivity = 78.26%, specificity = 69.57%), 0.8422 (95% CI: 0.7314–0.9530, sensitivity = 82.61%, specificity = 69.57%) and 0.7684 (95% CI: 0.6266–0.9102, sensitivity = 78.26%, specificity = 65.22%), respectively.

### 3.10 Differential exosome-derived miRNA target gene prediction and GO and KEGG enrichment analyses

**Figure 7:**
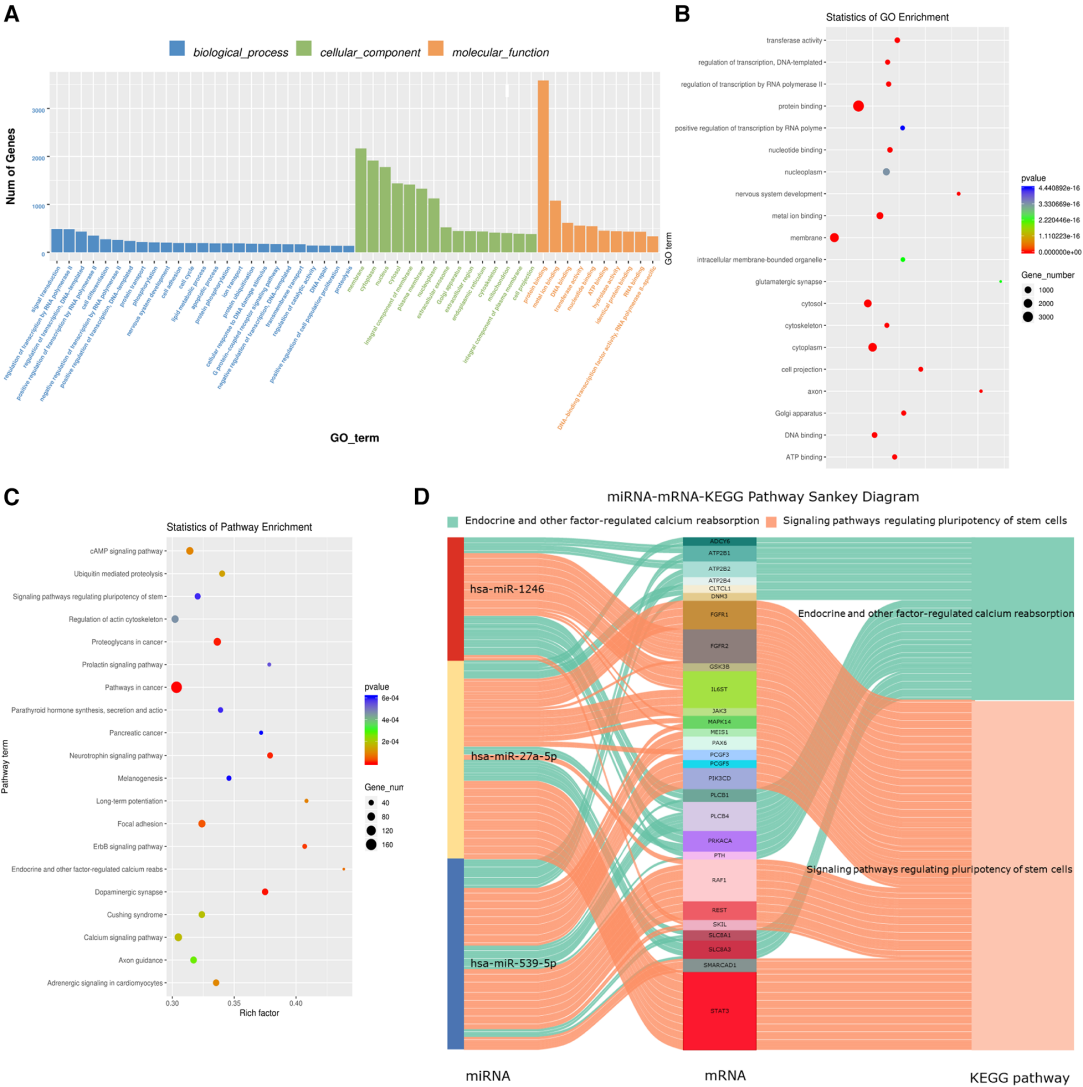
AIS plasma-specific exosome-derived miRNA target gene prediction and enrichment analysis. (**A**) GO enrichment results are categorized according to biological process, cellular component and molecular function. (**B**) GO enrichment Top 20 bubble chart. (**C**) KEGG enrichment Top 20 bubble chart. (**D**) “Endocrine and other factor-regulated calcium reabsorption” and “Signaling pathways regulating pluripotency of stem cells” pathway miRNA-mRNA-KEGG Pathway Sankey diagram.

Target gene prediction was conducted for hsa-miR-27a-5p, hsa-miR-539-5p, and hsa-miR-1246, followed by GO and KEGG enrichment analyses of the predicted target genes. First, the GO enrichment results were categorized and displayed according to biological processes, cellular components, and molecular functions (Figure 7A). The three exosome-derived miRNAs were primarily involved in two biological processes: signal transduction and regulation of RNA polymerase II transcription. They mainly belonged to cellular membrane and cytoplasmic components, and their main molecular function was related to protein synthesis. The top 20 results from GO enrichment and KEGG enrichment are displayed in bubble charts (Figures 7B,C). Among the top 20 enriched KEGG pathways, two pathways of interest were identified: “Endocrine and other factor-regulated calcium reabsorption” and “Signaling pathways regulating pluripotency of stem cells.” A miRNA-mRNA-KEGG pathway Sankey diagram (Figure 7D) was created using the genes of interest with significant flow in the two pathways. The majority of the genes shown to be regulated in this diagram were directed towards the “Signaling pathways regulating pluripotency of stem cells” pathway. Specifically, hsa-miR-27a-5p primarily regulated this pathway through STAT3, hsa-miR-539-5p mainly regulated it through RAF1, and hsa-miR-1246 regulated it through FGFR1 and FGFR2. Additionally, both hsa-miR-1246 and hsa-miR-27a-5p jointly regulated IL6ST, which had an impact on this pathway. These results suggest that the dysregulation of the plasma exosome-derived hsa-miR-27a-5p, hsa-miR-539-5p, and hsa-miR-1246 may be associated with the pathogenesis of AIS.

## 4 Discussion

AIS is the most common type of spinal curvature disorder, and screening for scoliosis in children and adolescents is of significant importance. The Scoliosis Research Society recommends that children undergo scoliosis screening at least 1–2 times at the ages of 10 and 12 to allow for early detection and intervention (30). SSS is considered an effective screening method that helps in the early detection of AIS and in timely intervention, potentially reducing the need for surgical treatment (31–33). This survey conducted through the SSS found that the overall prevalence of IS among 7- to 19-year-olds in Yunnan Province is 1.10%, which is slightly higher than the national prevalence rate (1.02%). However, it falls within the range of prevalence rates observed in different provinces in China (ranging from 0.11% to 2.64%) (9, 10, 34, 35). Research indicates that the prevalence of AIS is associated with various factors, such as ethnicity, altitude of residence, and socioeconomic status (6, 35). The prevalence of AIS varies among countries, including the United States (0.52%) (8), Japan (0.87%) (36), Saudi Arabia (0.78%) (37), India (0.61%) (33), Korea (0.497%) (38), and Iran (0.62%) (39).

This study reveals that in Yunnan Province, the incidence rate of IS stands at approximately 1.10%. Furthermore, a notable sex disparity was observed, with females exhibiting a higher prevalence of IS, being approximately 1.5 times more susceptible than males. Nevertheless, there was no statistically significant difference in the proportion of individuals with a Cobb angle ≥40° between the two sex. The prevalence of IS in Yunnan Province was found to be lower than that in certain regions, such as the Qinghai-Tibet Plateau, which is characterized by a high-altitude environment and a predominantly Tibetan population; However, the prevalence of IS in Yunnan Province showed a similar trend to previous studies in other ethnic groups and regions in China (6, 34, 35). Furthermore, it was observed that the incidence of IS exhibited a gradual rise in correlation with advancing age, peaking in the 13–14 years age range, followed by a subsequent gradual decline. Similarly, the Cobb angle also exhibited a similar trend, with the peak Cobb angle occurring at an age of 15 years. There may be two reasons for this trend: first, adolescents in this age group, especially girls, are in their growth spurt, and the rapid growth in height leads to a higher prevalence and rapid progression of scoliosis; second, adolescents in this age group experience a greater burden of schooling, and habits such as sedentary lifestyle, insufficient sleep and insufficient exercise increase the risk of scoliosis (20) This study also revealed that the IS population in Yunnan Province exhibited lower body weight and sitting height compared to the normal population. Moreover, the prevalence of IS varied across different educational stages, with the highest prevalence observed among middle school students. However, there was no significant difference in underweight prevalence between the two groups, which is consistent with findings from the study conducted by Scaturro and colleagues (40). Middle school students are in their growth spurt phase, and they also face significant academic pressure and reduced physical activity. This combination contributes to the higher prevalence of IS in this age group. In addition, among the 929 identified scoliosis patients, the distributions of severe scoliosis cases in males and females were similar. Among the single-curve scoliosis patients, thoracolumbar curves were the most common, and right-sided curves were more prevalent than left-sided curves. Furthermore, it was observed that there were more cases of double-curve scoliosis than single-curve scoliosis among the screened individuals. The diversity in the direction of IS scoliosis remains unclear but may be associated with asymmetrical activity of the muscles around the spine and compensatory curves (41). Some research suggests that right-handed dominance may be more likely to lead to rightward spinal curvature (42, 43).

The interaction between environmental factors and epigenetics may contribute to the imbalance in spinal growth and development, leading to the occurrence of AIS. While genetic factors play a significant role in AIS, they cannot fully explain the differences observed among patients. For example, identical twins with AIS may have no genetic differences, yet they can exhibit variations in the progression of spinal curvature. This suggests that epigenetics play an important role in the pathogenesis of AIS (44). Exosomes are a type of vesicle secreted by cells that serve as communication vehicles between cells. They contain a significant amount of miRNA. In recent years, increasing research has indicated that exosome-derived miRNAs, as epigenetic factors, are associated with bone metabolism (44–46). Exosome-derived miRNAs play a role in regulating processes related to the development of the musculoskeletal system, including embryonic development, osteoblast differentiation, bone marrow mesenchymal stem cell osteogenesis, paraspinal muscle differentiation, and cartilage formation., and they are directly or indirectly involved in the development of AIS (47–49). Additionally, exosome-derived miRNAs are protected by the outer vesicle, allowing them to remain stable in the RNA enzyme-rich environment of the blood (50, 51). Therefore, plasma exosome-derived miRNAs hold promise as specific molecular markers for the early screening of AIS. However, there is currently a lack of relevant research on the role of exosome-derived miRNAs in AIS.

AIS is a disease with an unpredictable and challenging natural history. Determining which patients require conservative or surgical treatment can be challenging. Early brace treatment can help reduce the progression of the curvature, improve aesthetic appearance, and decrease the need for surgery. Therefore, AIS screening before the Cobb angle exceeds 40° is of significant importance for conservative treatment (52, 53). To date, many experiments have verified the feasibility of exosome-derived miRNAs as markers for the diagnosis or treatment of diseases such as cancer (54), osteoporosis (55), osteoarthritis (56), and ankylosing spondylitis (57); however, studies on exosome-derived miRNAs in AIS patients are currently lacking. This study identified hsa-miR-27a-5p, hsa-miR-539-5p, and hsa-miR-1246 as key exosome-derived miRNAs associated with AIS, and verified that they have potential diagnostic value for AIS. Furthermore, José et al. (58) identified differentially expressed hsa-miR-27a-5p among AIS plasma miRNAs and validate its potential diagnostic value for AIS. Notably, the expression of plasma exosomal hsa-miR-539-5p was upregulated in sequencing data but downregulated in the RT-qPCR validation results in AIS patients in our study, which may be due to the bias caused by high-throughput sequencing and the small number of sequenced samples. However, due to the small number of high-throughput sequencing samples and relatively low accuracy, the results should be based on RT-qPCR validation results. In the future, more samples are still needed for validation regarding the diagnostic value of hsa-miR-539-5p in AIS.

The occurrence of AIS is associated with factors such as vertebral dysplasia, intervertebral disc development, neuromuscular conditions, and genetic regulation. However, these factors do not fully explain AIS (1). This study, through target gene prediction and pathway enrichment analysis, identified that hsa-miRNA-27a-5p, hsa-miRNA-539-5p, and hsa-miRNA-1246 are associated with two pathways: calcium metabolism and the regulation of stem cell differentiation. Skeletal growth and the maintenance of bone homeostasis are highly dependent on calcium metabolism and the differentiation of bone cells, particularly osteoblasts and osteoclasts (59). Furthermore, studies by Li et al. (60) found that extracellular vesicle-derived miR-27a-5p is related to bone formation. Research by Gautvik et al. (61) also showed that miR-27a-5p is associated with bone density. Pepe et al. (55) discovered that extracellular vesicle-derived miR-1246 is linked to osteoclast differentiation. Tripathi et al. (62) found that miR-539 is related to the regulation of osteoblastogenesis. Based on this evidence, it is inferred that the aberrant expression of hsa-miRNA-27a-5p, hsa-miRNA-539-5p, and hsa-miRNA-1246 in AIS patients may be associated with the progression of AIS. However, further exploration of regulatory mechanisms and functions is needed in the future.

In summary, this study was the first epidemiological survey of scoliosis conducted within all Yunnan Province, and the study cohort constructed by screening was further screened and validated for AIS-related plasma exosome-derived miRNAs, which provided new ideas and methods for the diagnosis and treatment of AIS. However, this study has certain limitations. Due to the economic disparities and uneven population distribution in most areas of Yunnan Province, information related to population distribution, economic conditions, lifestyle habits, etc., was not collected. Future work will focus on improving scoliosis surveys in remote areas and further analysing the regional differences in relevant risk factors. Additionally, the samples collected for exosome-derived miRNA sequencing and validation in this study were all from severe AIS patients. Future research will involve validation and analysis in patients with varying degrees of scoliosis from different regions and explore the functions and mechanisms of differential exosome-derived miRNAs.

## 5 Conclusions

This study was based on the SSS program conducted in Yunnan Province, China, and involved epidemiological screening for scoliosis in children and adolescents aged 7–19 years. The overall prevalence of IS was found to be 1.10%, and approximately 1.5 times higher in females than in males. The peak age for IS was 13 years, and there were more cases of double-curve deformities than single-curve deformities. At the same time, based on the established AIS research cohort, specific plasma-derived exosome-derived miRNAs associated with AIS were identified, including hsa-miR-27a-5p, hsa-miR-539-5p, and hsa-miR-1246. These exosome-derived miRNAs hold the potential to serve as molecular markers for diagnosing AIS and may be associated with the progression of the condition.

## Data Availability

The datasets presented in this study can be found in online repositories. The names of the repository/repositories and accession number(s) can be found below: https://www.ncbi.nlm.nih.gov/, Series GSE235203.
