# Supplementary material for: Prevalence and plasma exosome-derive microRNA diagnostic biomarker screening of adolescent idiopathic scoliosis in Yunnan Province, China
Source: Front Pediatr. 2024 Apr 24;12:1308931. doi: 10.3389/fped.2024.1308931 (PMC11076730; doi:10.3389/fped.2024.1308931)
Supplement: Supplementary file 1 [file Datasheet1.pdf]

Supplementary Material

1 Supplementary Table

Supplementary Table 1: Primer and Reference Sequences

Primer	Sequence (5'-3')	Length(bp)
hsa-miR-1246	GCCAATTTTTTGGAGCAGG	22
hsa-miR-27a-5p	AGGGCTTAGCTGCTTGTGA	19
has-miR-20a-5p	GCCGTAAAGTGCTTATAGTGCA	25
has-miR-20b-5p	GCAAAGTGCTCATAGTGCAGGT	24
hsa-miR-454-3p	GCCGGGCTAGTGCAATATTGCT	22
hsa-miR-539-5p	GCGGAGAAATTATCCTTGGTGT	24
hsa-miR-26a-5p	CGGCTTCAAGTAATCCAGGATA	25

2 Supplementary Figures

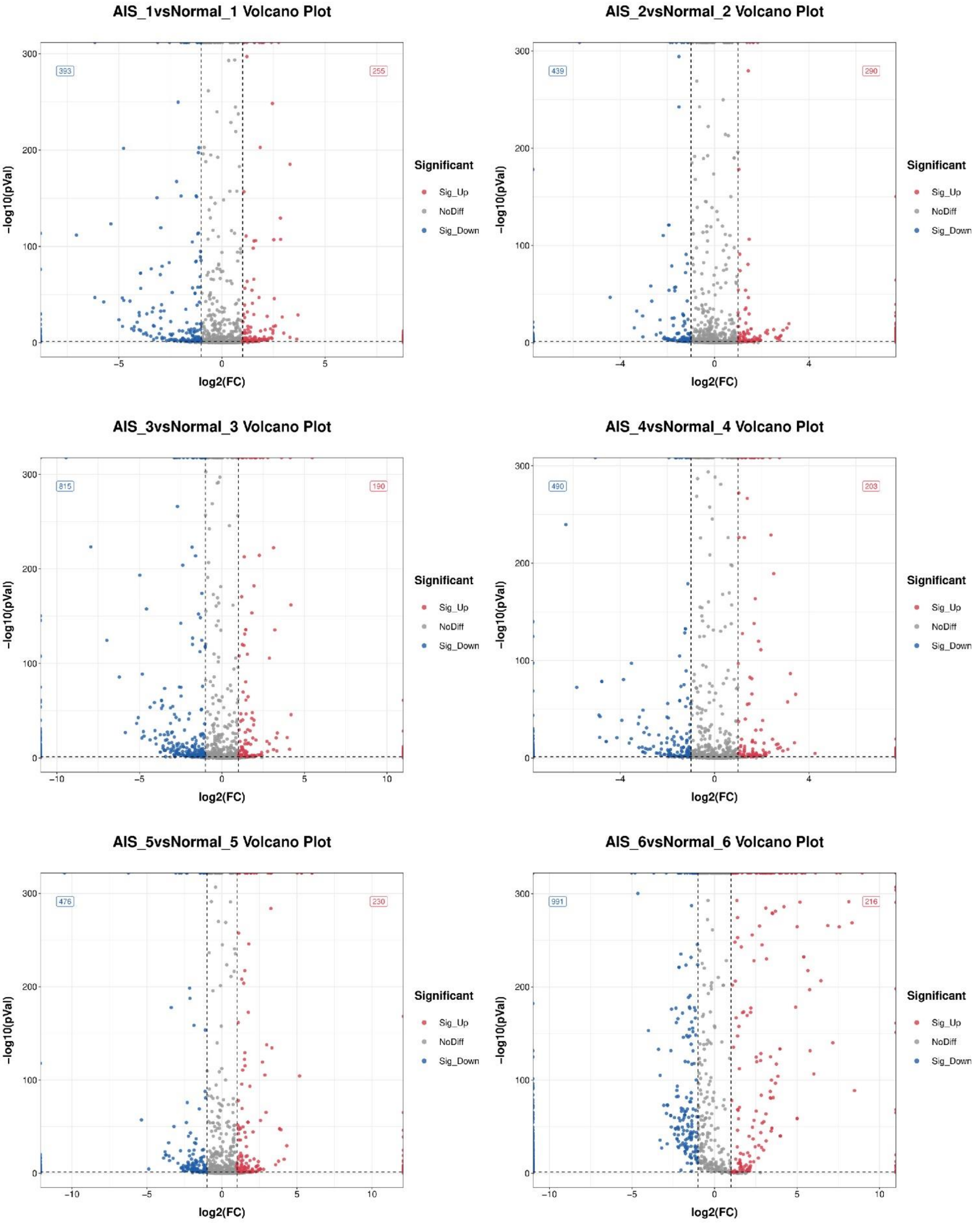

Supplementary Figure 1. The volcano plots for differential exosome-derived miRNA expression between the AIS and normal groups are shown as follows (***P*** < 0.05, |log2(FC)| ≥ 1). AIS_1 vs.

Normal_1: 255 upregulated and 393 downregulated miRNAs; AIS_2 vs. Normal_2: 290 upregulated and 439 downregulated miRNAs; AIS_3 vs. Normal_3: 190 upregulated and 815 downregulated miRNAs; AIS_4 vs. Normal_4: 203 upregulated and 490 downregulated miRNAs. AIS_5 vs. Normal_5: 230 upregulated and 476 downregulated miRNAs; AIS_6 vs. Normal_6: 216 upregulated and 991 downregulated miRNAs.
